# Enhanced Predictability of Urea Crystallization by an Optimized Laser Repetition Rate

**DOI:** 10.1021/acs.cgd.3c01210

**Published:** 2024-04-22

**Authors:** Leon Geiger, Ian Howard, Neil MacKinnon, Andrew Forbes, Jan G. Korvink

**Affiliations:** † Institute of Microstructure Technology, Karlsruhe Institute of Technology, Eggenstein-Leopoldshafen 76344, Germany; ‡ School of Physics, University of the Witwatersrand, Johannesburg 2017, South Africa

## Abstract

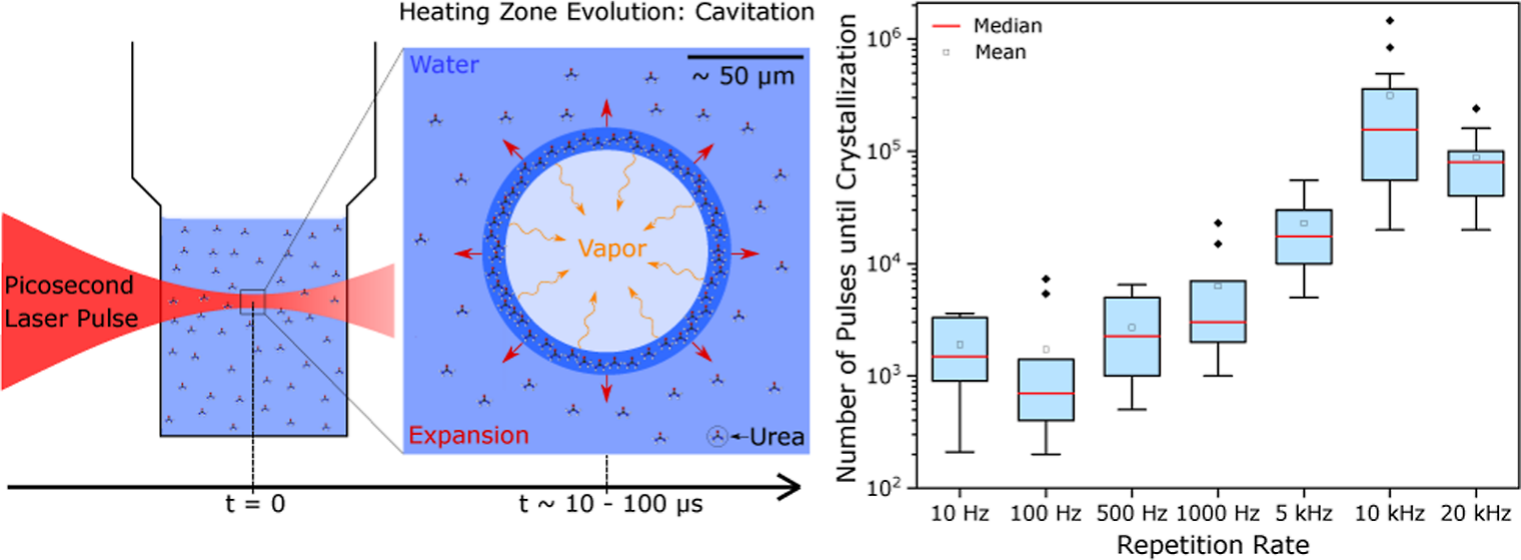

Laser-induced crystallization is a novel alternative to classical methods for crystallizing organic molecules but requires a judicious choice of experimental parameters for the onset of crystallization to be predictable. This study investigated the impact of the laser repetition rate on the time delay from the start of the pulsed laser illumination to the initiation of crystallization, the so-called induction time. A supersaturated urea solution was irradiated with near-infrared (λ = 1030 nm) laser pulses of pulse duration τ = 5 ps at a pulse energy of approximately *E* = 340 μJ while varying the repetition rate from 10 to 20,000 Hz. The optimal rate discovered ranged from 500 Hz to 1 kHz, quantified by the measured induction time (median 2–5 s) and the mean probability of inducing a successful crystallization event (5 × 10^–2^%). For higher repetition rates (5–20 kHz), the mean probability dropped to 3 × 10^–3^%. The reduced efficiency at high repetition rates is likely due to an interaction between an existing thermocavitation bubble and subsequent pulses. These results suggest that an optimized pulse repetition rate can be a means to gain further control over the laser-induced crystallization process.

## Abstract 2

The effects of the laser repetition rate on the induction of crystallization are investigated. Crystallization is detected within seconds in a supersaturated urea-D_2_O solution.

## Introduction

The crystallization of small molecules and proteins is an important technique, relevant in the structural determination process of proteins, purification of molecules, and overall pharmaceutical research. Furthermore, organic crystals are in great demand for new technologies in materials science and are already in the consumer market. To keep up with the increasing importance of organic crystals, research into fast and reliable crystallization methods is required. New challenges must be addressed through the combination of spatial and temporal control of the process, and laser-induced crystallization offers a route to such spatiotemporal control.

Laser-induced crystallization brings a known but not yet commonly used method to the crystallization toolbox. The classical methods for delicate organic molecules like proteins are based on evaporation, cooling, or precipitation with additives.^1−3^ The combination of these three methods is often used and must be optimized individually for each molecule. A significant disadvantage of these methods is the waiting time until a crystal emerges,^3^ and the associated probability of getting one at all. Furthermore, the addition of additives such as salts and precipitants adds further parameters that must be screened to generate the desired crystals. The laser-induced method is advantageous here since the nucleation is induced by a transient and switchable effect, the pulsed laser.

Laser-induced crystallization can be distinguished between two main regimes, depending on the laser intensity. Laser intensities of ∼MW/cm^2^ are classified as nonphotochemical-laser-induced-nucleation (NPLIN).^4^ To induce the nucleation, unfocused, nanosecond pulsed laser sources are used to interact with the molecule by the optical Kerr effect,^5,6^ the dielectric polarization,^7^ or by impurity heating.^8^ The second regime, related to NPLIN, is the laser trapping-induced crystallization (LTIC). In LTIC, a focused continuous-wave laser is used to increase the molecule concentration in the focal spot. In this process, the scattered light transfers momentum to the molecule in the direction of the spatial light gradient (i.e., the focal spot) among the force in the direction of light propagation.^4,9^

The applied process in this paper falls under the first regime of high-intensity laser-induced-nucleation, using a laser intensity of *I* = 10^14^ W/cm^2^. These high intensities are achieved by focusing a pulsed laser of femto- to nano-second pulse length in the sample solution. The energy is converted to heating of the water by multiphoton absorption, causing thermocavitation. The cavitation bubble drives an increased concentration at the bubble shell, leading to the nucleation. This mechanism has been known but has only been partially described theoretically.^10^ The same principle is used in sonocrystallization, where cavitation bubbles are created with ultrasound.^11^

Pulsed laser-induced crystallization of organic molecules has been known since 1996 by Garetz et al.^5^ and has been advanced in the early 2000s by Okutsu et al.^12,13^ and Yoshikawa et al.^14,15^ Mainly small molecules,^16−19^ amino acids,^20^ and small proteins^15^ have been crystallized with pulsed lasers so far.

Laser-induced crystallization using the pulse train output from regenerative amplifiers is a process started by a cavitation bubble which is formed by the heat input of the high instantaneous power laser pulse in a small volume.^10,21,22^ One critical figure of merit for laser-induced crystallization is the induction time, the amount of time that elapses between the start of the pulsed laser irradiation and crystal formation. Previous work has examined how the pulse energy^14^ and pulse length^23^ affects laser-induced crystallization. It was found that pulse lengths in the picosecond range were optimum. No optimum has yet been found for the pulse energy; the rule of thumb here is that the higher the better.^6,14,23^ In this previous work, amplifiers with a 1 kHz repetition rate were used.^14,23^

This work explored whether the induction time could be reduced by simply increasing the repetition rate of the laser. Varying the repetition rate from 10 Hz to 20 kHz and measuring the induction time for at least 10 urea solutions at each repetition rate allow the probability of induction to be estimated (as a function of the repetition rate) and the mean induction time for each repetition rate to be found. It was observed that the induction time decreased minimally at repetition rates greater than 500 Hz. The induction time staying relatively constant despite the significant increase in the repetition rate indicates that the probability of induction at higher repetition rates must be reduced. This is hypothesized to be related to the lifetime of the cavitation bubble created by a pulse, measured to be on the order of 100 μs.^15,16,22,23^ When the inverse of the repetition rate exceeds this lifetime (10 kHz), it becomes likely that the cavitation bubble from a preceding pulse is still present when a subsequent pulse arrives. This could mean that the pulse interacts with the existing cavitation bubble rather than creates a new one, thereby lowering the probability of induction. The cavitation bubble lifetime would therefore limit the repetition rate until the induction time is reduced and above which it no longer changes.

## Experimental Setup, Materials, and Methods

### Materials

Sample solutions consisted of crystalline urea (CH_4_N_2_O, analysis grade) dissolved in deuterium oxide (99.9% D_2_O). A 50 mL volumetric flask was used to prepare a stock sample solution of 12.25 M urea. The solution was kept at 60 °C for 1.5 h to ensure solubility, and then, 6 mL was filled into the sample cuvettes with a syringe (50 mL volume) and syringe-filter (pore size 0.8 μm) before sealing with a cap. The samples were allowed to cool slowly to 20 °C overnight in a polystyrene foam box. Eight samples were produced with one solution batch of 50 mL: one sample for each repetition rate plus an eighth as a backup-sample in the case of spontaneous crystallization in the cooling process (details summarized in Table 1 in the Supporting Information). The 12.25 M urea concentration was chosen because it was the highest concentration which remained a metastable supersaturated solution during cooling, and handling did not cause spontaneous crystallization (urea solubility in pure water at 21 °C is 109.6 g/100 g,^24^ corresponding approximately to a 9.7 M solution). It was assumed the solubility of urea in D_2_O was the same as for H_2_O. Urea crystallization experiments have been reported using 800 nm pulsed lasers in water solutions.^14,23^ To maintain a similar level of light absorbance with our near-infrared laser, deuterated water (D_2_O) was used.^25^ A similar cavitation bubble evolution in deuterated water is assumed due to the small difference in the boiling point.^26^

The cuvette was a modified polystyrene vessel with plane sides (Brand Vial Coulter Counter PS with a PE Cap). Flat faces were chosen to eliminate incoming laser beam refraction. The vessel was modified by drilling a hole in one side and gluing a microscope coverslip over the hole, glass being more compatible with the high power of the laser (the unmodified plastic cuvette melted at average laser powers of 340 mW and above, equivalent to 1 kHz repetition rate and higher).

### Laser System

**Figure 1:**
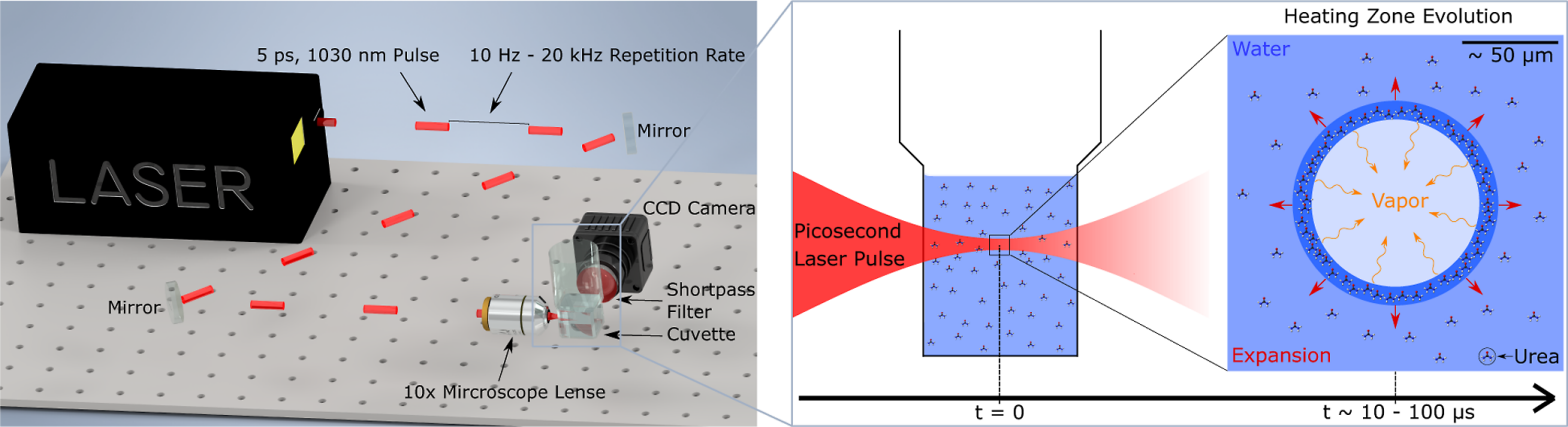
Left: schematic representation of the pulsed laser setup. In the laboratory, additional mirrors were used to direct the light, different to the setup shown. Due to reflection losses at each of the mirrors, the measured power in front of the objective was 344 mW at 1 kHz. Right: process of laser-induced nucleation. The laser-illuminated region induces a volume filled with vapor. The bubble expands due to heating during the pulse, growing until equilibrium is reached. The formation of a crystal nucleus is triggered by the increased concentration at the liquid–vapor interface.^10^

A modular femtosecond laser system (Light Conversion Pharos) with a center wavelength of 1030 nm was used for this work. It has a tuneable pulse length, repetition rate, and pulse energy. For the experiment, the pulse length was set to 5 ps full width at half-maximum (FWHM) and the energy per pulse to 400 μJ. The measured average power of the beam at a repetition rate of 1 kHz, before it enters the 10×-microscope lens, was 344 mW, corresponding to a pulse energy of 344 μJ. The maximum laser output energy was chosen to reduce the stochastic influence of pulse energy on crystallization.^14^ Test experiments with temporal pulses from 270 fs to 5 ps FWHM showed that 5 ps is most promising for inducing urea crystallization, and thus, this was used for all subsequent experiments. The laser system can provide repetition rates from 1 Hz to 20 kHz. This range was divided in seven values with the selected frequencies of 10, 100, and 500 Hz and 1, 5, 10, and 20 kHz. The test experiments showed no crystallization within 10 min with 1 Hz which is why this frequency is not included in the experiments. The 10× infinity-corrected microscope lens focused the 5 mm beam down to a diameter of roughly 4 μm. The full setup is shown in Figure 1.

Crystallization was monitored by recording each experiment with a simple webcam camera, observing through the clear sidewall of the cuvette (5 fps and 720p resolution). The induction time was determined by the time between frames when illumination started and when a crystal became visible. The number of pulses required to induce crystallization could be calculated from the pulse rate and induction time. The assumed error of the induction time is 2 frames, which translates into 0.4 s. Within these 2 frames, the detection delay between the nucleation and a detectable crystal size is included. The growth rate of the (001) growth mode is simulated with 0.02 nm/ns.^27^ The detectable size with this setup is ≥ 1mm which is smaller than the size of a crystal growing for 0.4 s. From the distribution of the number of pulses required to induce crystallization, the probability of induction is extracted (see the Supporting Information for a detailed explanation of the evaluation). The maximum illumination time was 10 min; if crystal induction was not observed in that time, then nucleation was deemed unsuccessful. Each experimental round was completed within 2 h. The experiments were performed in a temperature and humidity-controlled laboratory at 20 °C and 50–60% relative humidity.

## Results and Discussion

**Figure 2:**
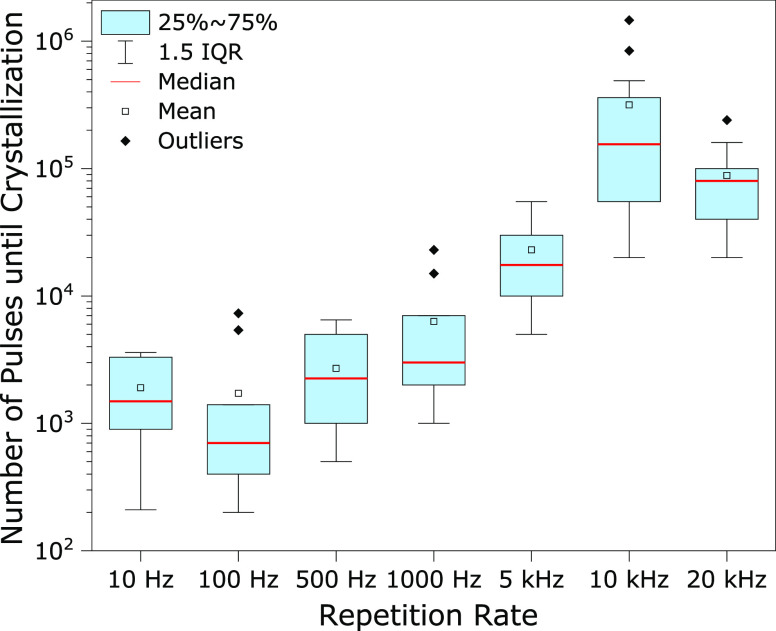
Number of laser pulses to induce crystallization at the different repetition rates (double logarithmic representation). The number of pulses needed to achieve a successful crystal increase with the repetition rate, suggesting an inhibitory effect at the higher rates. Only successful crystallization events are plotted, data are summarized in Tables 1 and 2 in the Supporting Information.

The objective of this study was to determine the influence of the laser pulse repetition rate on the urea crystallization induction time, assuming that the crystallization occurred within the 10 min cutoff window. Urea was chosen as a model system to study due to its previous use in laser-induced crystallization^5,14,23^ and because of its similarity to amino acids, which is helpful in regard to protein crystallization and drug discovery. The number of pulses required to induce crystallization generally increases with the repetition rate, as presented in Figure 2 (data summarized in Tables 1 and 2 in the Supporting Information). The plot can be divided into low and high repetition rate regimes. In the low repetition rate regime (i.e., 10 to 1000 Hz), the median number of pulses needed to induce crystallization increased slowly (ranging between 100 and 10 000 pulses). In the high repetition rate regime (i.e., above 1 kHz), the number of required pulses significantly increased, suggesting that a higher number of pulses per second actually hinders the process of induction.

To explain the observed crystallization dependence on the pulse repetition rate, we consider the effects of a pulse generating a cavitation bubble, together with the influence of the immediately following pulse. The cavitation bubble mechanism has been described in previous laser-induced crystallization experiments^10,14−16^ and is shown schematically in Figure 1. A laser pulse induces local heating and water vaporization via multiphoton absorption of the infrared laser light, forming a cavitation bubble. The size and lifetime of the bubble are dependent on the laser energy and pulse length. For saturated urea solutions at comparable laser pulse length, bubble diameters of up to 150 μm and lifetimes of 22 μs have been reported.^23^ As the bubble expands, the local solute concentration at the bubble surface increases, and a “successful” pulse results when the first molecules organize into a crystal lattice, forming the first seed crystal. The two-step nucleation theory dictates that the increased local concentration first leads to a prenucleation cluster featuring partial molecular orientation encouraged by the increased local density.^28,29^ This cluster forms the basis for the seed crystal, which forms in the second step.

Depending on the pulse rate, a subsequent laser pulse has the chance to interact with the seed crystal (or prenucleation cluster), the already formed bubble, or both. In the case of the pulse impacting the oriented molecules (seed crystal or cluster), the energy input is likely large enough to overcome the intermolecular forces and redissolve the cluster. If the pulse interacts with an already formed bubble, local turbulence may be introduced, which disturbs the bubble and suppresses the nucleation mechanism. The introduction of turbulence was observed in our experiments: at low pulse repetition rates, bubbles were transported vertically, as would be expected in a static fluid. However, at increasing rates, the bubbles traveled significant distances horizontally, either toward or away from the focal spot (see Figure S1).

There is a distinctive irregularity at 10 kHz, where the average number of pulses required to induce crystallization is a factor of 10 greater than would be expected from the general trend (Figure 2). At 10 kHz, the time between pulses (*t* = 1/10,000 Hz = 100 μs) matches closely to the cavitation bubble lifetime of laser-induced crystallization (tens of microseconds^15,22,23^ to 100 μs^16^) including the seed crystal or cluster formation (assuming that the seed crystal or cluster formation happens within the bubble lifetime because only during that time, the conditions are suitable). In the case that molecular orientation is achieved by a pulse, the next incoming pulse arrives as the cavitation bubble collapses and thus has a high probability of destroying the association. At lower repetition rates, the seed crystal may have sufficient time to move away from the focal point, so that it does not directly interact with the next pulse. While the 20 kHz repetition rate also falls within the range of potential bubble and nucleation lifetimes, we observe a slightly higher probability of crystallization compared to 10 kHz. It could be that under our experimental conditions, the bubble lifetime coincided with the 10 kHz pulse rate, and that this measurement offers an indirect measurement of the nucleation time. We refer to this as repetition rate antiresonance since the net result is a reduced probability of crystallization. We note that more insights could be drawn by using a high speed camera; unfortunately, this was not an option in our experiment.

**Figure 3:**
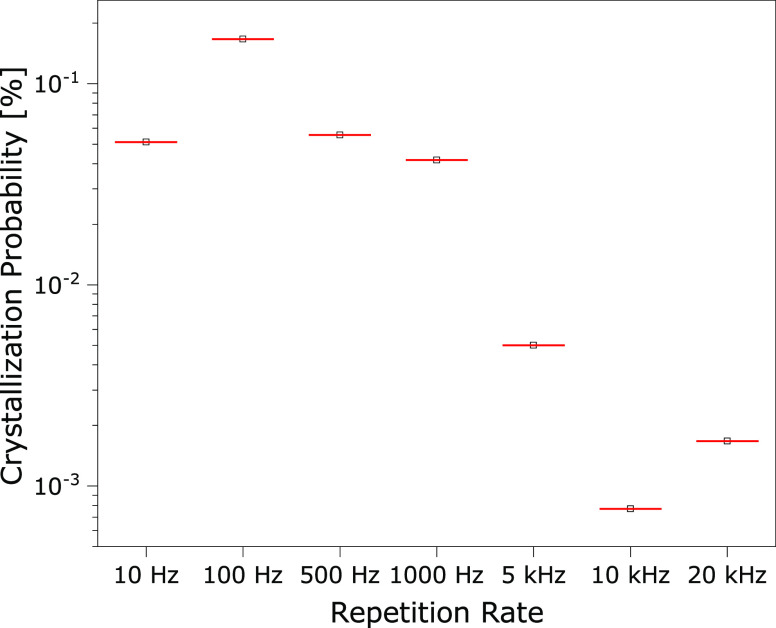
Plotted probability to start the crystallization, based on the fitting of the CDF. All PDF and CDF histograms are attached to the Supporting Information Figures S2–S8.

Using the data from the induction time versus repetition rate experiments, it is possible to extract the probability of inducing crystallization using a cumulative distribution function (CDF) (see Figure 3). The CDF representing the total probability that crystallization has not been induced after *N* pulses, given the probability of successful induction is *p*, is (1 – *p*)^*N*^. Thus, the CDF giving the probability that crystallization has been initiated within *N* pulses is 1 – (1 – *p*)^*N*^. The full set of probability density functions (PDF) and CDF for each repetition rate can be found in the Supporting Information (Figures S2–S8). A student’s *t*-test analysis of the number of required pulses between successive repetition rates revealed no statistical difference at rates from 10 to 1000 Hz (Bonferroni correction applied to the *p*-value, significance tested at α = 0.05). Therefore, the data were grouped into low regime (10–1000 Hz) to increase statistical significance, while the data in the range from 5 to 20 kHz were examined individually. In the group for the lower repetition rates, the best fitting probability is 1/1900. Examining the CDF, a 50% probability that induction has already occurred is reached after 1400 pulses, and the probability reaches 90% after 4400 pulses. In comparison, the probability for 5 and 20 kHz is an order of magnitude lower with 1/20 000 and 1/60 000, with 14 000 and 42 000 pulses for 50% probability that induction has already occurred, and 47 000 and 139 000 pulses for 90% probability. In the repetition rate antiresonant case at 10 kHz, the probability goes down to 1/130 000, with 91 000 pulses for 50% probability that induction has already occurred, and 300 000 pulses for 90% probability.

**Figure 4:**
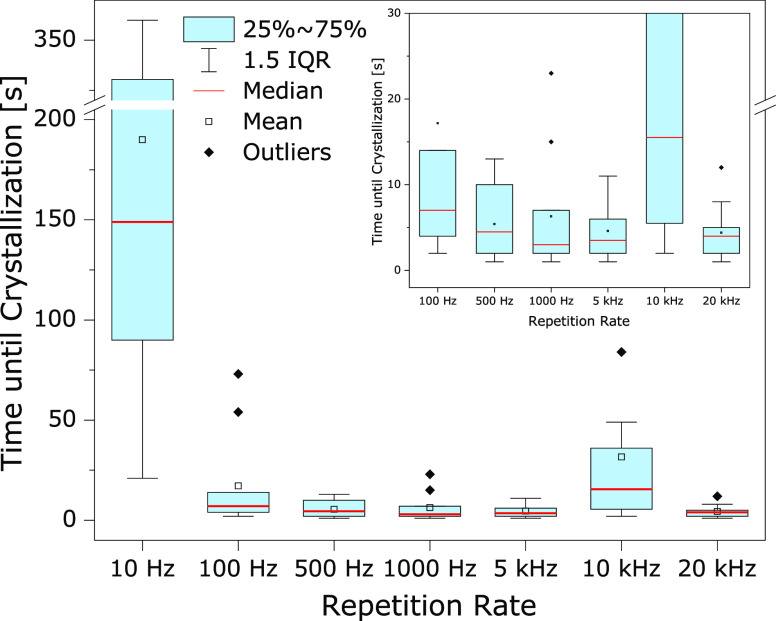
Plot of the elapsed times until the onset of successful crystallization. A particular experiment is considered successful if crystallization occurred within 10 min. Inset: enlarged view of 100 Hz to 20 kHz.

These results demonstrate that higher repetition rates do reduce the induction time as compared to lower repetition rates, but once 500 Hz is passed, further increasing the repetition rate is no longer beneficial. This is reflected in the observations that (i) the total time before crystallization initiated was reduced with increasing repetition rate and (ii) the variation in induction time decreased with the increasing repetition rate (Figure 4, Table 2 in the Supporting Information).

## Conclusions

It has been shown that the repetition rate has an effect on the induction time. The average time needed to induce crystallization decreases with an increasing repetition rate. Several minutes are required at 10 Hz, which reduces to seconds at 500 Hz to 1 kHz. Thus, almost instantaneous crystallization is possible. However, decreasing the induction time below this point is not possible by further increasing the repetition rate. The probability of inducing crystallization drops significantly at higher repetition rates, with the results revealing that a lower repetition rate has up to 250 times higher probability of inducing crystallization than higher repetition rates. At 10 to 1000 Hz, the mean probability of inducing crystallization is 0.05%. In the worst case of antiresonance matching, the probability reduces to 0.0008%.

Therefore, the optimum conditions for inducing crystallization are a compromise. A lower repetition rate is favorable due to less general disturbance of the crystallization process by a subsequent pulse, but a high repetition rate clearly has the advantage of the faster rate of pulse arrival to trigger the crystallization. The sweet spot seems to lie in the range of 500 Hz to 1 kHz, fortuitously well-matched to the repetition rate of many standard amplifier systems.

In terms of the mechanism for reducing the probability of induction at high repetition rates, each subsequent pulse into the solution can destroy the nucleation conditions created with the first pulse. If the cavitation bubble, the prenucleation cluster, or the seed crystal is disturbed, crystal growth will be hindered. This is anticipated in the case of higher repetition rates, where there is a greater chance for such interfering effects being brought into the system.

One particular effect is the repetition rate antiresonance. In this case, the pulse interval is on the same time scale as the development time of the cavitation bubble and nucleation. The next laser pulse may arrive when nucleation is most likely. This inhibition of nucleation was observed in the number of pulses until crystallization at 10 kHz, falling outside the general trend, corresponding well to previous observations of cavitation bubble lifetimes on the order of 100 μs.^16^

In summary, with induction times on the second time scale, laser-induced crystallization can offer good temporal control over the initiation of crystallization. However, the probability of inducing crystallization, even in the optimum 500–1000 Hz repetition rate regime, remains low. Further work to study how improved control of the optical pulses could increase the probability of induction remains of clear interest. Ultimately, the goal of reliably inducing crystallization with only a single pulse is worth continued exploration.

## Supporting Information Available

Details of growth patterns; evaluation of probability density and CDF; and discussion on heat input, solubility, convection, and crystal growth (PDF)
