# Supplementary material for: Enhanced Predictability of Urea Crystallization by an Optimized Laser Repetition Rate
Source: Cryst Growth Des. 2024 Apr 22;24(9):3589–94. doi: 10.1021/acs.cgd.3c01210 (PMC11066841; doi:10.1021/acs.cgd.3c01210)
Supplement: Supplementary file 1 — cg3c01210_si_001.pdf [file cg3c01210_si_001.pdf]

Supplementary Information

Enhanced predictability of urea crystallization by optimized laser repetition rate

Leon Geiger,[†] Ian Howard,[†] Neil MacKinnon,[*,†] Andrew Forbes,[‡] and Jan G. Korvink[*,†]

[†]*Institute of Microstructure Technology, Karlsruhe Institute of Technology, Eggenstein-Leopoldshafen, Germany*

[‡]*School of Physics, University of the Witwatersrand, Johannesburg, South Africa*

E-mail: neil.mackinnon@kit.edu; jan.korvink@kit.edu

Supplementary Figures and Tables

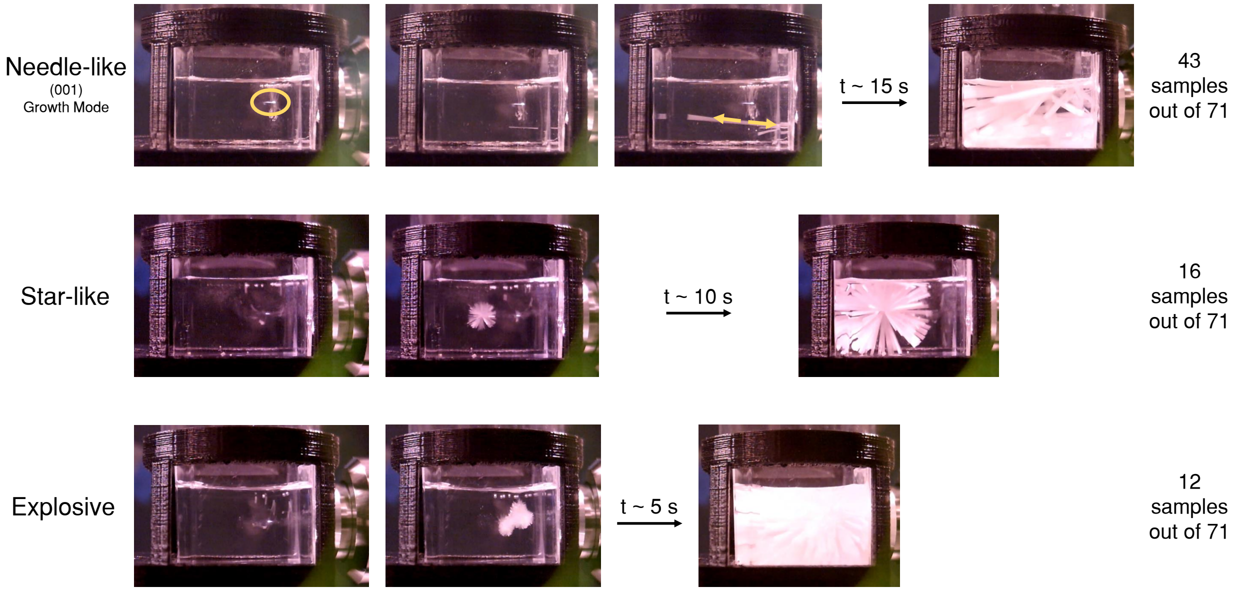

Figure 1: Growth patterns observed during laser induced crystallization. The laser focus is exemplary marked in the upper row. For the needle-like growth the bi directional growth is highlighted. Here also the movements of the generated gas bubbles can be seen, which lead to the mixing of the solution.

Table 1: Successfully induced crystallization experiments per repetition rates occurred within 10 min. In some cases, more than ten data points were measured because the 8th-backup sample of one batch was used. The solutions of the 10 Hz measurements were reused if no crystallization occurred after 1 h after the first illumination:

Repetition Rate [Hz]	Number of Samples	Successfully Induced Crystallization	Percentage of Crystallized Solutions [%]
10	18	8	44.4
100	11	11	100
500	10	10	100
1k	10	10	100
5k	10	10	100
10k	12	12	100
20k	11	10	90.9

Table 2: Detailed summary of experiments, excluding unsuccessful experiments:

Repetition Rate [Hz]	Average Number of Pulses until induction	Median of Number of Pulses	Average Time to crystallize [s]	Median Time to crystallize [s]
10	1900 ± 4	1490 ± 4	190 ± 0.4	149 ± 0.4
100	1720 ± 40	700 ± 40	17.2 ± 0.4	7 ± 0.4
500	2700 ± 200	2300 ± 200	5.4 ± 0.4	4.5 ± 0.4
1k	6300 ± 400	3000 ± 400	6.3 ± 0.4	3 ± 0.4
5k	23000 ± 2000	17500 ± 2000	4.6 ± 0.4	3.5 ± 0.4
10k	316000 ± 4000	155000 ± 4000	31.6 ± 0.4	15.5 ± 0.4
20k	88000 ± 8000	80000 ± 8000	4.4 ± 0.4	4 ± 0.4

Evaluation of probability density and cumulative distribution functions

The geometric function is a type of exponential function, used by assuming that the probability of nucleation reduces with every pulse. These include the effects of heating the solution due to the energy input, convective fluid movements causing turbulence and, as the repetition rate increases, the direct influence of the following pulse.

For these measurements, at least 10 runs per repetition rate were performed. The exponential decay is not evident in some distribution plots. Especially at low repetition rates, the mentioned influences (heating, convection, influence of the following pulse) are small and

therefore one could also assume a constant probability. To remain constant in the evaluation, all distribution functions were assigned to the geometric distribution function. In Section 3 of the SI, the total heating of the solution and convection are examined in more detail. Towards high repetition rates, the disturbing influence of the following pulses on the nucleation probability is clear. As described in the main text, the bubble lifetimes and nucleation times become equal to the pulse intervals.

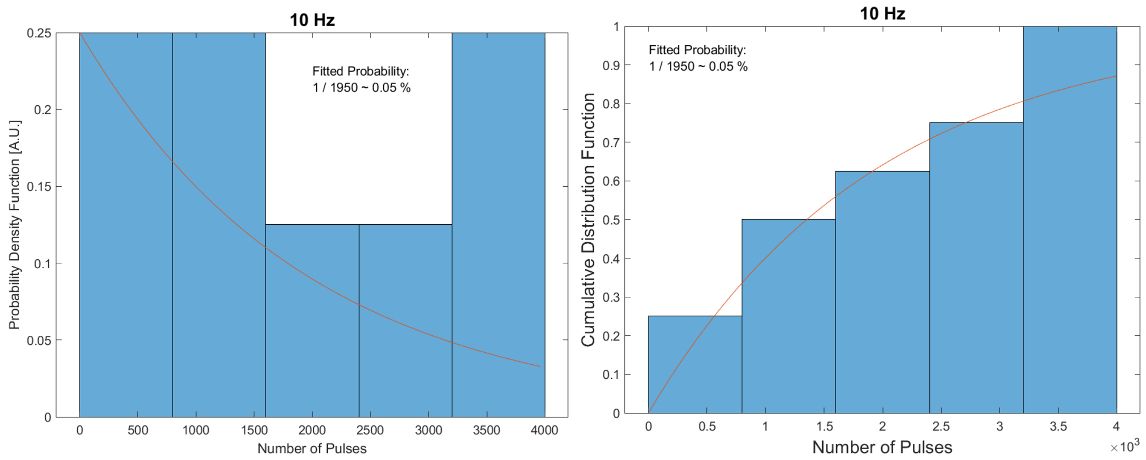

Figure 2: Probability density and cumulative distribution function fitted with the estimated probability of 1/1950 for the 10 Hz measurements. The best fits are shown as red lines. Sturge's Rule was used to select the number of bins: $1 + 3.322 * log_{10}(N)$, where N is the number of experiments per repetition rate. The result is 4.3 which was rounded to 5 to avoid over-smoothing.

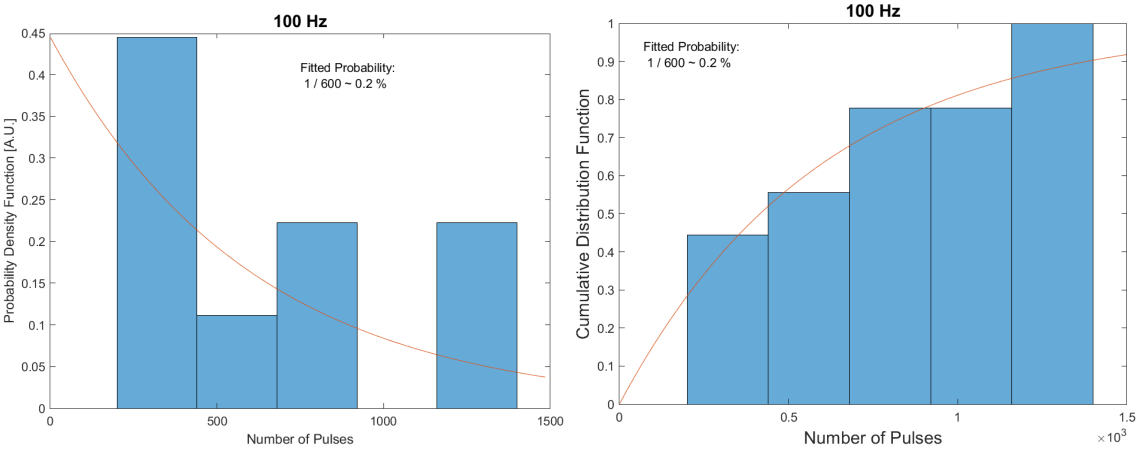

Figure 3: Probability density and cumulative distribution function fitted with the estimated probability of 1/600 for the 100 Hz measurements. The best fits are shown as red lines.

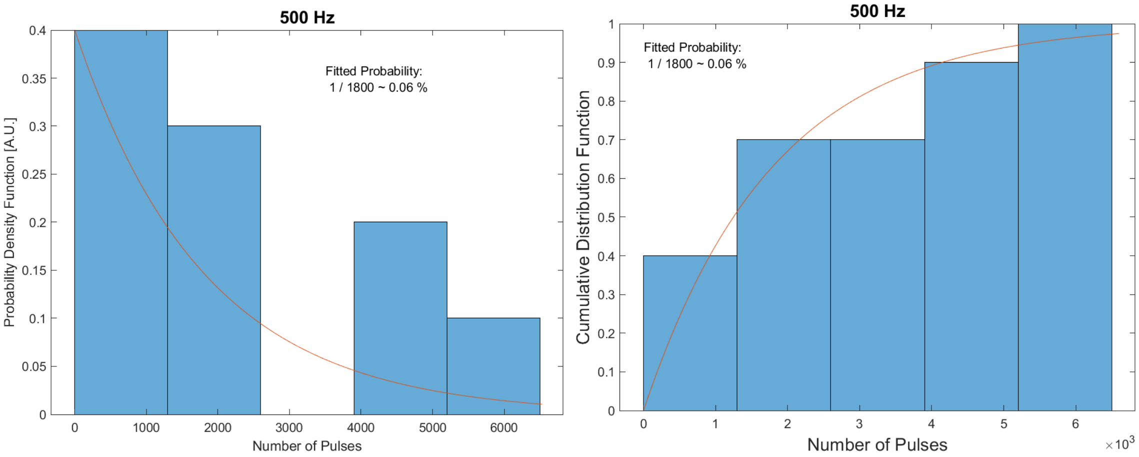

Figure 4: Probability density and cumulative distribution function fitted with the estimated probability of 1/1800 for the 500 Hz measurements. The best fits are shown as red lines.

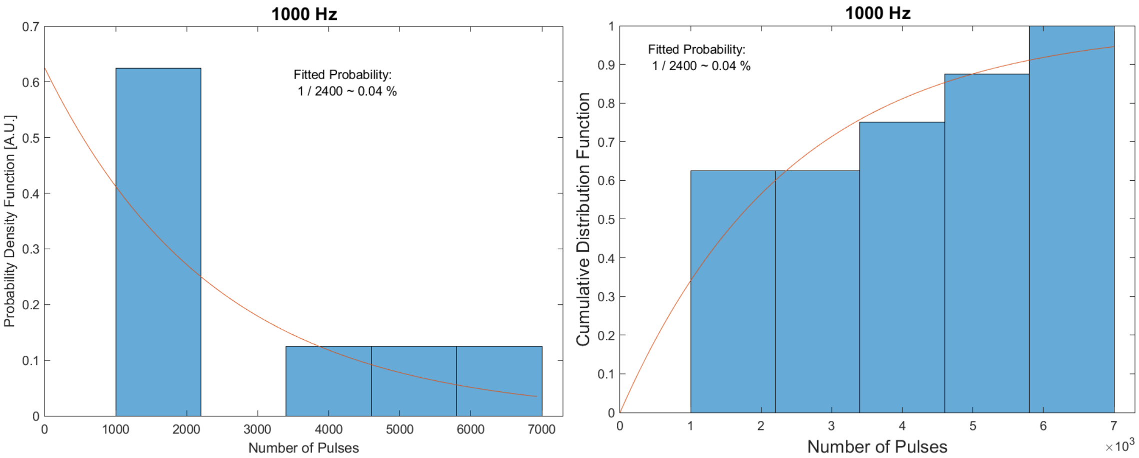

Figure 5: Probability density and cumulative distribution function fitted with the estimated probability of 1/2400 for the 1000 Hz measurements. The best fits are shown as red lines.

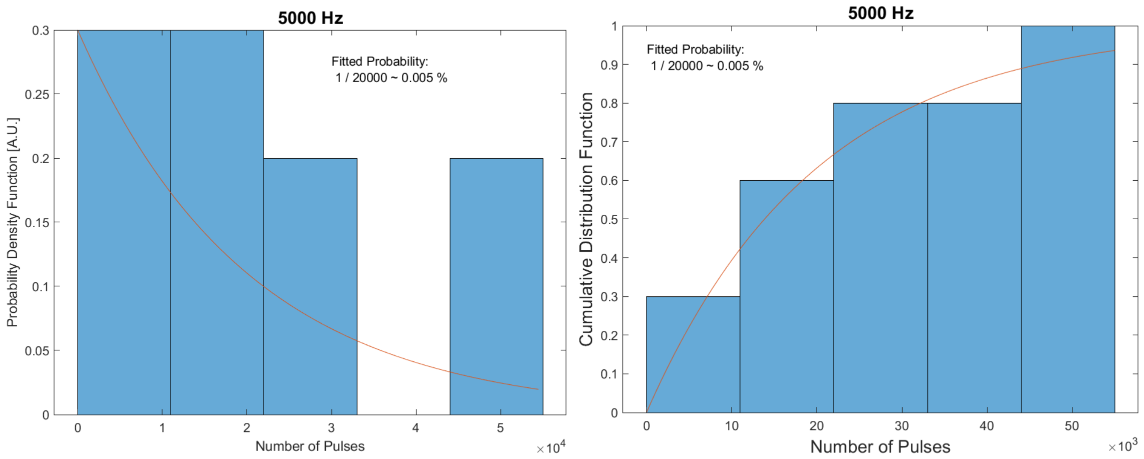

Figure 6: Probability density and cumulative distribution function fitted with the estimated probability of 1/20 000 for the 5000 Hz measurements. The best fits are shown as red lines.

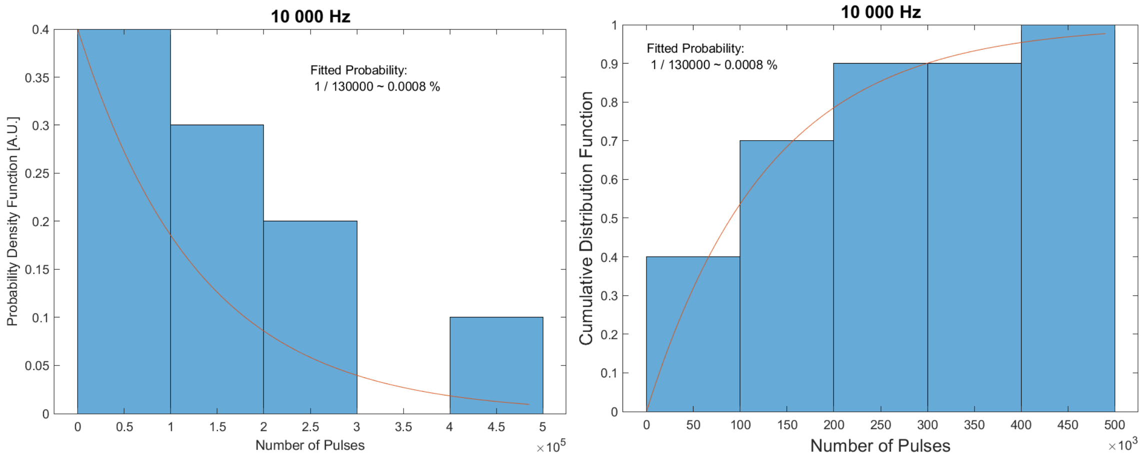

Figure 7: Probability density and cumulative distribution function fitted with the estimated probability of 1/130 000 for the 10 000 Hz measurements. The best fits are shown as red lines.

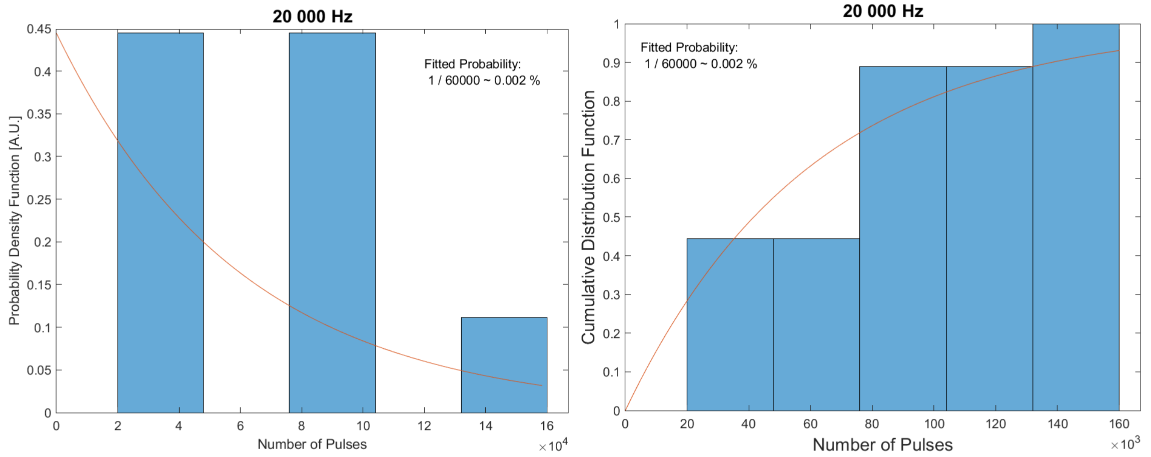

Figure 8: Probability density and cumulative distribution function fitted with the estimated probability of 1/60 000 for the 20 000 Hz measurements. The best fits are shown as red lines.

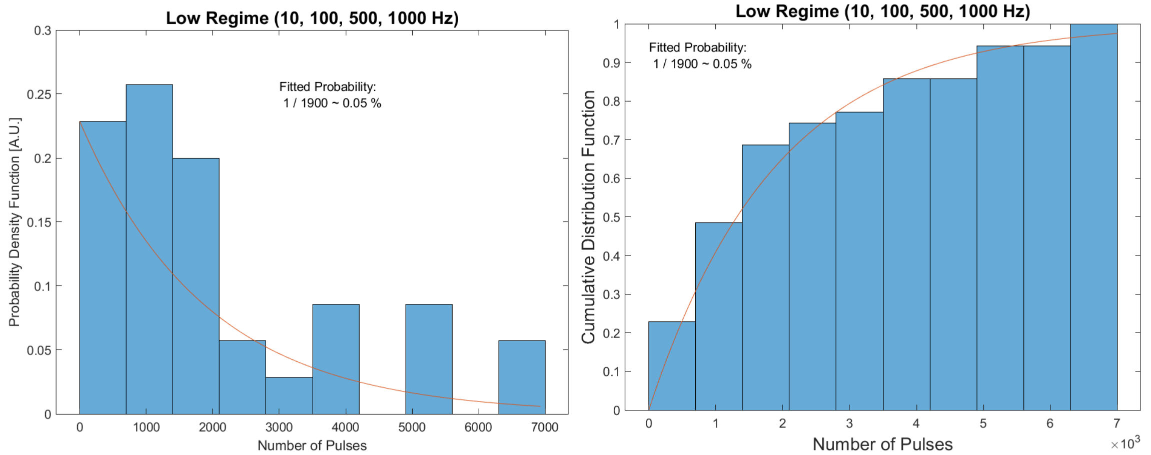

Figure 9: Probability density and cumulative distribution function fitted with the estimated probability of 1/1900 for the combined data using repetition rates from 10 - 1000 Hz. A Student's t-test analysis comparing the the number of pulses used between successive repetition rates showed no significant difference between the data sets from 10 Hz to 1000 Hz (p-values Bonferroni corrected for multiple comparison, significance tested at $\alpha = 0.05$). Therefore these data were grouped into a 'low' regime (10 - 1000 Hz) to increase statistical significance.

Further Results

Heat Input and Solubility

As high power laser pulses with high repetition rates were used, the influence of laser power on the solution temperature was examined. Figure 10 shows the measured temperature evolution and calculated solubility changing over time at different repetition rates. For the temperature measurement a cuvette was filled with 6 mL deuterium oxide. Due to challenges in measuring temperature and calculating solubility, simplifications were made. A pure deuterium oxide instead of the 12.25 M urea solution was used for the temperature measurements because a saturated urea solution will crystallize during temperature measurements as the temperature probe acts as an crystallization site. The calculated solubility is derived from urea-water (H_2O) solution[1] because data for the solubility of urea in deuterium oxide D_2O are not available. Studies comparing the crystallisation of an organic molecule in water and deuterium oxide could also not be found. One paper was found on the crystallization of a cooled propanediol solution, where there were no major differences in the kinetics.[2] Hence, it was assumed that the solubility in water and deuterium oxide is the same. Despite these approximations, the data shown in Figure 10 is useful for qualitative discussion. It can be seen that for repetition rates up to 1 kHz the system needs 10 minutes to reach thermal equilibrium. For 5, 10 and 20 kHz thermal equilibrium is not reach within 10 minutes. Especially the temperature gain of 18 °C at 10 kHz (even more for 20 kHz) creates a significant increase in solubility. After a few minutes at these high repetition rates - respectively 5 min for 20 kHz and 7.5 min for 10 kHz - the calculated solubility of urea in water exceeds the concentration of the 12.25 M solution. This means that more urea would be soluble; however, in all experiments in this work at high repetition rates, crystallization was already completed after a few seconds. The measured outliers at 10 kHz, which took up to 2.5 minutes, may be slightly influenced by the rising temperature hindering crystallization. At 1 kHz and lower repetition rates we do not expect a significant effect on crystallization due to the slight temperature increase of up to 2 degrees. The temperature

increase for 10 Hz was not measurable and therefore not plotted in the graph. In summary, the observations indicate that temperature changes of the entire solution do not affect the induction of crystallization at any repetition rate.

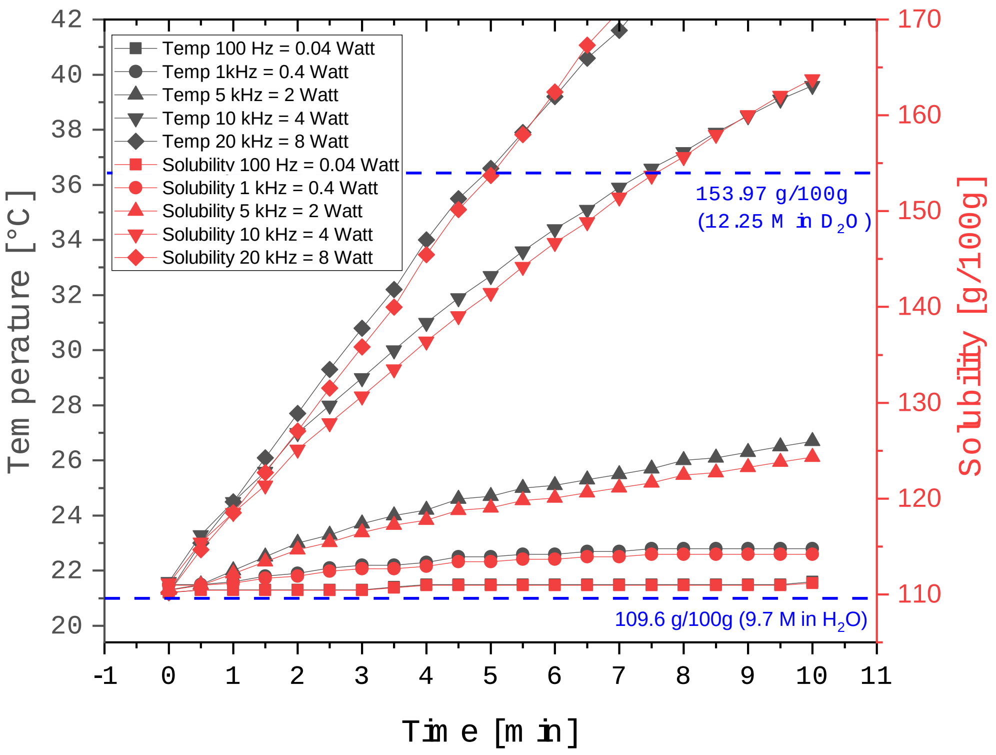

Figure 10: Measurement of pure deuterium oxide temperature change with the different laser repetition rates. The cuvette was filled with 6 mL. Added are the corresponding solubility curves for water. This plot demonstrates the relevance of temperature effects on the solubility. When the solubility exceed the concentration (blue lines for 12.25 M and 9.7 M crystallization will not occur due to the full solubility of the Urea in the solvent. All experiments with 12.25 M solutions and high repetition rate induced crystallization in a short time frame before complete solubility was reached. Solubility data in pure water from.[1]

Convection

Convection was observed in the experiments. During the laser irradiation, gas bubbles originated from the focus area and traveled upwards. The higher the repetition rate, the more convection and turbulent flow occurred, with the flow is directed horizontally from the laser focal point. Seed crystals are ejected from the focus like the bubbles. On the one hand, this

leads to a permanent mixing of the solution, which has advantages in heat equalization and therefore provides a fresh solution in the laser focus. On the other hand, convection leads to a disturbance of the environment for both cavitation bubble growth and seed crystal formation.

Types of crystal growth

The type of crystallization was also investigated by evaluating each experimental video. In the Table 3 one can see how the crystal growth evolved from the point at which it became visible in the camera footage. It was categorized in the four types of crystal growth which were present in the experiments. The most common type is a single needle-like structure which growth in two opposite direction. From Singh[3] it became clear that the growth is dominant along the (001)-face which defines the needle-like structure. Also growth occurs on the (110)-face but with a slower growth rate. The (110)-face growth is responsible for the thickness of the needle. These growth modes can be observed (Figure 1), where a fast needle growth and a slow thickness growth is visible. After the grown needle bounces of the cuvette wall the crystal grows in different directions until the whole cuvette is filled with crystal structures. For the needle-like type the total growth time is up to a minute.

Less common is several needle-like structural growth in different positions or a star like growth from on seed crystal. Possible causes of growth of these several needle-like structures are to find in the focus of the laser. For example, an already induced seed crystal by pulse one can be hit by another pulse and ablate fragments. These fragments grow independently and merge together at an early stage of growth. This phenomena allows crystal growth with a few needle-like growth sides (several needle- and star-like). The ongoing convection inside the cuvette can either help to push away the seed crystal from the focus, but also it can bring the seed back into the focus. Hence, there is a certain cross section for the seed to be hit again.

The last category is the explosive growth. It is a extreme form of the last two categories. As seen in Figure 1 the visible growth directly starts as a cloud of multi-orientated crystal.

The polymorphous appearance suggests that ablation on a seed crystal produces many small fragments that grow independently in different directions. This explosive growth type fills the whole cuvette in a few seconds with the crystal structure. Overall, the growth of crystals structures from pure needle-like to explosive is a continues structural transitions, i.e. mixed growth states like several-needle types occur as well.

Interestingly there is a difference in the time to resolve the urea crystals for the purpose of cleaning the cuvette. After adding few milliliters of pure water to the laser-induced crystal structures dissolve much quicker than the crystals which were unintentionally grew while cooling the samples over night. Also unintentionally grown crystals are optical transparent. Nevertheless the laser induced crystals grow needle-like, which indicates a single crystal structure, the opaqueness of it indicates a less compact lower quality crystal. Presumably this is related to the crystal growth rate, as it is generally assumed that slower growth improves crystal quality.[4,5] The laser-induced crystals grow faster - in seconds - and therefore suffer from a less compact structure which makes them opaques.

In summary, a direct correlation between the repetition rate and the type of crystal growth was not found. Due to the fast crystal growth after induction a more opaques crystal grew. The most common type is a single needle-like crystal. That other types also occur is probably due to the interaction of an already formed seed crystal, which is disturbed by further laser pulses and broken into fragments (ablation). Overall, the conditions in focus are very difficult to investigate. For a accurate control of the crystallization a single pulse induction is desirable so the mentioned effects are less disturbing.

Table 3: Categorization of the different types of crystallization growth:

Repetition Rate [Hz]	Single needle-like	Several needle-like	Star-like	Explosive
10	4	1	1	2
100	8	1	0	2
500	6	2	1	1
1k	6	3	0	1
5k	8	0	1	1
10k	5	3	1	3
20k	6	2	0	2
Total	43	12	4	12

References

(1) Lee, F.-M.; Lahti, L. E. Solubility of urea in water-alcohol mixtures. *Journal of Chemical & Engineering Data* **1972**, *17*, 304–306.

(2) Mehl, P. M. Suppression of crystallization in the binary system 1,2-propanediol-deuterium oxide. Comparison with light water as solvent. *Thermochimica Acta* **1995**, *255*, 297–317.

(3) Singh, M. Simulating growth morphology of urea crystals from vapour and aqueous solution. *CrystEngComm* **2015**, *17*, 7731.

(4) McPherson, A.; Gavira, J. A. Introduction to protein crystallization. *Acta Crystallographica Section F Structural Biology Communications* **2013**, *70*, 2–20.

(5) Wlodawer, A., Dauter, Z., Jaskolski, M., Eds. *Protein Crystallography*; Springer New York, 2017.
